# Drought Drives Extracellular Polymeric Substances Accumulation and Functional Shifts in Streambed Biofilm Communities

**DOI:** 10.1007/s00248-025-02649-3

**Published:** 2025-11-13

**Authors:** Anna M. Romaní, Núria Perujo, Marta Pujol, Giulia Gionchetta

**Affiliations:** 1 GRECO, Institute of Aquatic Ecology, University of Girona, Girona, 17003 Spain; 2 Department of River Ecology, Helmholtz Centre for Environmental Research - UFZ, 39114 Magdeburg, Germany; 3 Department of Environmental Chemistry, Institute of Environmental Assessment and Water Research (IDAEA), Spanish Council of Scientific Research (CSIC), Barcelona, 08034 Spain

**Keywords:** Biofilm matrix, Microbial functional fingerprint, Intermittent streams, Drought, Prokaryotic communities, Heterotrophic functional diversity

## Abstract

This study investigates the adaptive response of streambed microbial biofilms to water scarcity, focusing on the role of extracellular polymeric substances (EPS) production across a gradient of hydrological conditions. Sediment samples from 37 streams in the north-eastern Iberian Peninsula, encompassing both permanent and intermittent flow regimes, were analysed for EPS-polysaccharide content, microbial biomass, chlorophyll-a, and biofilm function (carbon substrate utilization profiles). Drought conditions were characterized based on the number of dry days over the eight months preceding sampling. Results revealed that EPS production increased significantly in intermittent streams, particularly under long-term drought, reaffirming that EPS synthesis is a key microbial strategy to mitigate desiccation stress. Notably, when normalized to prokaryotic density, EPS content exhibited a significant positive correlation with drought duration, emphasizing the dominant role of heterotrophic bacteria over algae in EPS secretion. However, EPS content alone was not a universal indicator of water scarcity, which showed a large variability in permanently flowing streams. Functional profiling showed clear shifts in carbon substrate utilization associated with stream hydrology. Intermittent streams exhibited a broader metabolic range, and particularly a capacity to use phenolic compounds, suggesting an adaptation to terrestrial organic matter inputs. Contrary to expectations, functional diversity increased in drier conditions, challenging assumptions derived from controlled experiments and underscoring the resilience of Mediterranean microbial biofilm communities to drought. These findings provide empirical support for EPS-mediated drought adaptation in natural biofilms and highlight functional diversity as a potential mechanism maintaining ecosystem processes under increasing aridity due to climate change.

**Supplementary Information:**

The online version contains supplementary material available at 10.1007/s00248-025-02649-3.

## Introduction

Biofilms represent the predominant form for life for bacteria and archaea on Earth [1]. In the environment, biofilms are composed of aggregates of bacteria, archaea, algae, fungi and protozoa that adhere to surfaces. In aquatic ecosystems, biofilms primarily develop on rocks, cobbles and sediment particles where they play a fundamental role in the ecosystem’s metabolism, actively absorbing and transforming organic compounds and nutrients [2, 3]. Similarly, in soils, microbial biofilms are essential in stabilizing soil aggregates and regulating nutrient cycles [4]. Biofilms exhibit remarkable resistance to environmental stressors, such as salinity, drought or the presence of toxic compounds, earning them the designation of “a fortress” due to their resistance to external disturbances [5]. This resistance is largely attributed to the self-produced extracellular polymeric substances (EPS) matrix, which embeds biofilm microorganisms, providing structural integrity and stability [6]. The EPS matrix facilitates enzyme and nutrient sharing, retains toxicants, creates diverse ecological niches, and serves as a crucial protective barrier against desiccation. By maintaining a highly hydrated microenvironment around biofilm organisms, the EPS enhances desiccation tolerance in water-deficient environments [5].

Water scarcity is a crucial stress factor for biofilms and microbial life [7]. In many regions a decline in water availability has been observed, and climate change models predict a global decrease in precipitation and an increase in potential evapotranspiration, leading to heightened aridity [8]. Specifically in rivers and in the Mediterranean climate regions, drought episodes are expected to become longer, more intense and frequent [9]. Previous studies in streambed sediments indicate that prolonged drought can reduce prokaryotic viability in biofilms and induce a functional shift toward utilizing organic matter from terrestrial sources [10]. Additionally, long-term drought conditions have been associated with shifts in the composition of prokaryotic and algal communities and a decline in primary production [11, 12]. Beyond these functional and community-level changes, biofilms subjected to drought may undergo structural modifications, particularly in the development of the EPS matrix as a protective mechanism. However, the substantial energy investment required for EPS production (can be up to the 50%, the rest of energy allocated to growth and division [13, 14]), may compromise biofilm growth and function [15]. Experimental laboratory studies have demonstrated that simulated long-term drought (five months) leads to a gradual increase in EPS-polysaccharide content, coupled with a decrease in hydrolytic enzyme activity [16]. Similarly, in soils, drying conditions have been shown to increase EPS content as a stress response to water deficit [17]. A biofilm colonization study found higher EPS content in intermittent Mediterranean streams than in central European stream biofilms, suggesting that Mediterranean biofilms are more adapted to drought conditions [18]. This further supports the idea that microbial communities in drought-prone environments have evolved distinct strategies to cope with water scarcity, reinforcing the ecological significance of EPS production in fluctuating hydrological conditions.

Despite the recognized importance of EPS in environmental biofilms, field data on its role and adaptive mechanisms remain scarce [4]. Given climate change predictions, the role of EPS in environmental biofilms under drought conditions is a critical area of research which requires field-based studies. Here we aim to empirically assess whether biofilm EPS production increases under escalating water scarcity conditions and to explore its potential link to biofilm microbial functioning changes. To achieve this, streambed sediments were collected from 37 streams exhibiting a wide range of hydrological histories, from permanently flowing streams to those experiencing drought from 5 to 100% of the eight months preceding sampling. The polysaccharide EPS content of the biofilm was measured, along with carbon substrate utilization profiles as a proxy for biofilm function and functional diversity. We hypothesize that biofilm responses to water scarcity involve a trade-off between the production of EPS and the microbial functioning, dictated by the allocation of energy towards either metabolic activities or resistance strategies [15]. Under extreme drought conditions, the development of protective mechanisms to withstand desiccation—such as increased EPS production—is expected to come at the cost of biofilm functioning. Overall, this study provides empirical data on the structural and functional responses of microbial biofilms to drought in natural environments.

## Methods

### Study Approach

The sediment from 37 streams in the north-eastern Iberian Peninsula (Catalonia) was collected and stored either fresh or frozen, depending on the type of analysis. The sampling sites and strategy followed those of the previous study by Gionchetta et al. [10]. Briefly, these sites are located along the region’s main rivers and include both lowland and mid-mountain watercourses (< 1200 m above the sea level), which are submitted to significant variations in mean annual precipitation (350–1200 mm), seasonal intermittency with increasingly prolonged dry periods, and diverse land cover types, ranging from natural and semi-natural to anthropogenic (Suppl. Fig. S1). At each site, three surface sediment samples were collected, chosen randomly from a ca. 100 m length reach, using a methacrylate corer (5 cm deep, 4.5 cm in diameter) and transported to the laboratory under cold conditions (4 °C). Before the sampling, stream hydrology was monitored over an 8-month period (245 days) to characterize hydrological conditions. Based on the number of days without surface water during this period, the stream sites were classified in four hydrological categories: FL for flowing permanently sites (never dry sites, *n* = 8, note that we included here 1 site that had 2 days of drought at the beginning of the 245 days, and was hystorically a typicaly permanent flowing site so we decided to include it in the FL category), SD for short-dry sites (dry 11–41 days, 4.5–16% of time, *n* = 6), MD for medium-dry sites (dry 60–152 days, 24.5–62% of time, *n* = 16), and LD for long-dry sites (dry 189–245 days, 77–100% of time, *n* = 7) (Fig. S1). Sampling was performed in early autumn, in wet conditions, to homogenize the hydrological conditions between sites before sampling.

Microbial communities at the phylum level are dominated by Proteobacteria [10]. The most evident compositional shift among the categories studied is the decrease of Cyanobacteria from FL to all intermittent sites (with the lowest relative abundance in LD), accompanied by an increase of Bacteroidetes and candidate division WP2 (or Eremiobacterota), which are known to be abundant in several dry, bare soil environments [19].

### Microbial Functional Fingerprint

Sediment samples for the analysis of carbon substrate utilization profiles using Biolog EcoPlates were processed following the protocol described in [20]. Biolog EcoPlates contain 96 wells with 31 different carbon sources in triplicate and are used to analyze the metabolic fingerprints of microbial communities. Briefly, biofilm was detached from sediment particles by soft sonication minimizing cell lysis (2 times x 1 min in cold, sonication bath, Selecta, operating at 40 W and 40 kHz, following the same detaching protocol as for prokaryotic density, Supplementary Information SI1) and vortex (30 s at maximum speed) of suspensions containing 1 cm^3^ of sediment (approx. 1 g of sediment) in 5mL of pyrophosphate (final concentration 0.1 M) and then 1 ml of this extract was diluted in 9 mL of sterilized Ringer solution. Microplate wells were then inoculated with 130 µL of the diluted sediment extract under sterile conditions and incubated at 20 °C in the dark. Incubations were initiated 24–48 h after sampling. Absorbance readings were taken at 24, 48 and 168 h at 590 nm using a microplate reader (Tecan, Infinite M200 Pro 4). Raw absorbance data for each well were corrected by subtracting the mean absorbance of the control wells, and values below 0.05 were set to zero. Functional α-diversity (Shannon diversity index [21] was calculated from two incubation time measurements: from 24 h absorbance measurements, to capture the initial functional response from the fresh sample, and from the integrated absorbance values considering all time-points, to capture the potential functional response. The integrated absorbance was obtained from the entire curve evolution of absorbance for each substrate and integrate the area below this curve (multiplying the absorbance by the incubation time difference for each time point [22]),. To check for differences in the functional fingerprint between samples, data from all time-points were analysed by compositional data analysis (CODA) approach as described in [23] (see details in Data analysis). CODA is approriate for data like those obtained from BiologEcoplate results (based on relative color development between wells, dependending on the inculum and varying with incubation time). CODA is a quantitative description of the parts or components of a whole, conveying exclusively relative information between parts [24], and is based on the application of isometric log-ratios to avoid distorting the results with spurious correlations [25]. By the CODA approach the discrimination of potential differences in the carbon substrate utilization profiles from a data set is optimized also taking the advantage of including the whole data set (i.e. data from the different incubation time measurements) [23].

### EPS Polysaccharide Content, Prokaryotic Density and Chlorophyll-a Content

One sediment subsample (1 cm^3^ volume) for polysaccharide content in extracellular polymeric substances (EPS) was collected from each sediment corer using a plastic core created with a sterile uncapped syringe (1 cm diameter), frozen and stored at −20 °C prior to analysis. EPS was extracted from the sediments using a cation exchange resin (CER, Dowex Marathon C, sodium form, Sigma-Aldrich) following the procedure described in [26]. Polysaccharide content of EPS was measured by the spectrophotometric phenol-sulphuric assay [27] using a standard curve of glucose (0–100 µg/mL). Results, expressed as µg of glucose-equivalents per g of sediment dry weight (DW), were also normalized per prokaryotic density (EPS/Prokaryote) and chlorophyll-a content (EPS/Chl-a). Dry weight (DW) was measured by oven dry (70 °C, 72 h) an extra sediment sample (1 cm^3^) from each sediment corer.

For prokaryotic density, a sediment subsample (1 cm^3^ volume) was collected from each sediment corer. These subsamples were fixed with 10 mL of filter-sterilized synthetic water (0.2 μm pore size nylon filters, Whatman, Kent, UK) and formaldehyde (2% final concentration) and then stored at room temperature prior to prokaryotic density analysis. Prokaryotic density was determined by flow cytometry (FACSCalibur, Becton Dickinson) after disaggregating and cleaning the sample, and staining it with Syto13 (see the detailed protocol in the Supplementary Information SI1). Results are given as prokaryote cells per g of sediment DW.

For chlorophyll-a analysis, a subsample of 1.5 cm³ was collected from each sediment corer and kept frozen (−20 °C) until their analysis. For the pigment extraction, 10 mL of 90% acetone was added to each sample, and left covered overnight at 4 °C in the dark. The following day, the samples were sonicated (sonication bath, Selecta) for 4 min and the extracts filtered through GF/C glass fiber filters (1.4 μm pore size) to clarify them before the absorbance measurement. Absorbance scans from 400 to 800 nm wavelengths were performed (UV-1800 Shimadzu spectrophotometer) and the concentrations of chlorophyll-a calculated [28]. Throughout the filtration and measurement process, the extracts were handled at low temperatures and with minimal light exposure to prevent degradation, as chlorophylls are highly sensitive to these factors. Results are given as µg per g of sediment DW.

### Data Analysis

The analysis of the microbial functional fingerprints was performed on triplicates of the 37 samples (*n* = 111) following [23]. Briefly, absorbance data from Biolog Ecoplates were isometric log-ratio transformed and repeated measures MANOVA was conducted to assess differences in functional fingerprints across stream hydrological categories (between-subjects) and incubation time (within-subjects). Canonical variate plots facilitated visualization of group separation by hydrological category. Scores from the canonical variate axis 1 were extracted and served as a proxy for the microbial functional fingerprint.

One-way analyses of variance (ANOVA) were performed to test the presence of significant differences between the four stream hydrological categories (FL, SD, MD, and LD) for the biological variables analysed (EPS, prokaryotic density, chlorophyll-a, EPS/prokaryote, EPS/chl-a, Shannon-functional diversity). Variables were log-transformed to reach normality and homoscedasticity. Post hoc Tukey’s HSD test was used to distinguish between sampling stream categories when significant differences were obtained.

To analyse a potential gradual effect of stream intermittency to the biological variables, linear regressions were performed with the specific total dry days for each stream as independent variable. For this regression analysis, the CAN1 axes (from the canonical variate plot) was used as a proxy for the microbial functional fingerprint. The potential relationships between functional and structural responses (CAN1 vs. EPS and EPS/prokaryote) were also tested by linear regression analyses.

All analyses were performed either with the entire data set or by selecting the intermittent sites (discarding permanent flowing streams). All the analysis and plots were obtained using R software 4.4.3 [29] (*zCompositions*,* candisc*,* car*,* ggplot2* packages) [30–33].

## Results

### Microbial Functional Fingerprint Along the Hydrology Gradient

**Fig. 1:**
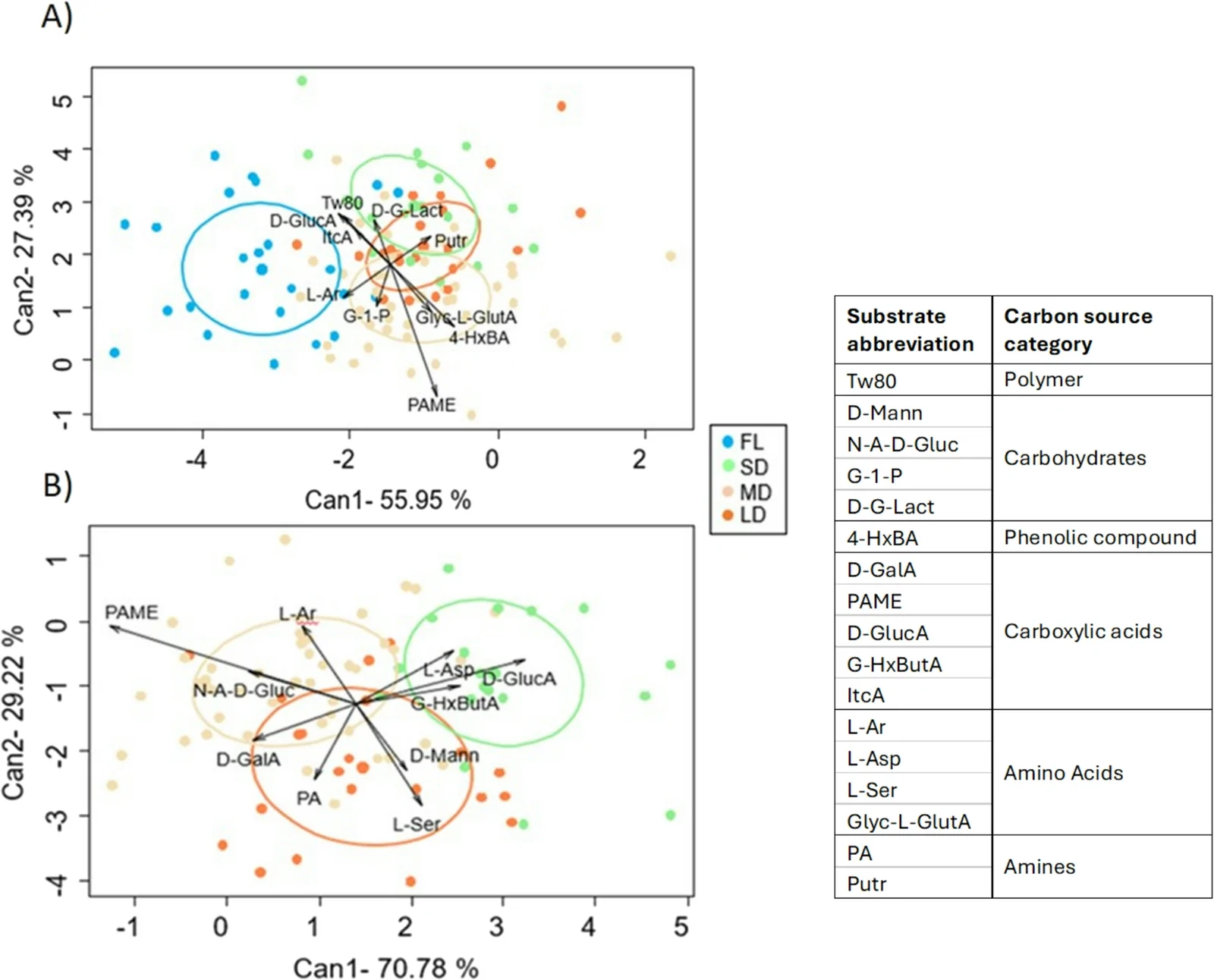
Canonical variate plots illustrating the microbial functional fingerprint based on carbon substrate utilization: **(A)** including all the stream categories, **(B)** including only intermittent streams. Colours represent the four stream categories: FL (Flow), SD (Short Dry), MD (Medium Dry), and LD (Long Dry). Dots correspond to the Biolog Ecoplate results at the different incubation time points for each sampling site. The legend includes the specific substrates appearing in the Figures; details of the 31 carbon substrate names and their respective carbon source category are shown in Table S1. Ellipses indicate the 95% confidence interval

Both hydrology and microplate incubation time had a significant effect on carbon substrate utilization profiles across stream categories (Table 1, repeated measures MANOVA analysis, Fig. 1). Furthermore, the influence of hydrology on the functional fingerprint was consistent for the different plate incubation time points as shown by the non-significant hydrology × time interaction (Table 1). Specifically, significant differences were observed between flowing (FL) streams and all intermittent streams (Fig. 1A; Table 1). Among intermittent streams, SD sites showed slight differences (although non-statistically significant) from MD and LD streams in carbon substrate utilization profiles (*p* = 0.08, Fig. 1B; Table 1). While no clear separation was observed between specific carbon substrates and stream categories, FL sediments tended to exhibit greater utilization of polymers (e.g. Tw80), carboxylic acids (D-GlucA, ItcA), and amino acids (L-Ar), whereas intermittent streams (SD, MD, and LD) showed a preference for utilization of phenolic compounds (e.g. 4-HxBA), and also amino acids and amines (Glyc-L-GlutA and Putr, respectively, Fig. 1A). When comparing intermittent sites, SD streams were mostly separated by the first axis from MD and LD streams (Can1, Fig. 1B) and main differences were due to the use of different types of carboxylic acids; SD streams displayed higher usage of the D-GlucA and G-HxButA, while MD and LD streams favoured the utilization of PAME and D-GalA (Fig. 1B). Furthermore, MD and LD streams were characterized by using carbohydrates (N-A-D-Gluc) while SD favoured the use of the amino acid L-Asp (Fig. 1B, Supl Table S1).

**Table 1:** Results of repeated measures MANOVA for the microbial functional fingerprint. The *p*-values are shown for the hydrological stream category (hydrology), the biolog Ecoplate incubation time (time), and the interaction hydrology x time effects. In **(A)** all the stream categories are included (i.e., FL (Flow), SD (Short Dry), MD (Medium Dry), LD (Long Dry)), whereas in **(B)** only the intermittent sites, SD, MD and LD, are included. Significance code is defined as: *** = *p* < 0.001, **= *p* < 0.01, *=*p* < 0.05,.=*p* < 0.1

	A) All categories	B) Only intermittent
Hydrology	0.0053**	0.0832.
Time	0.0003***	0.0073**
Hydrology x Time	0.3684	0.3183

### Effect of Hydrology History on EPS Production and Relationship To Biofilm Functioning

**Fig. 2:**
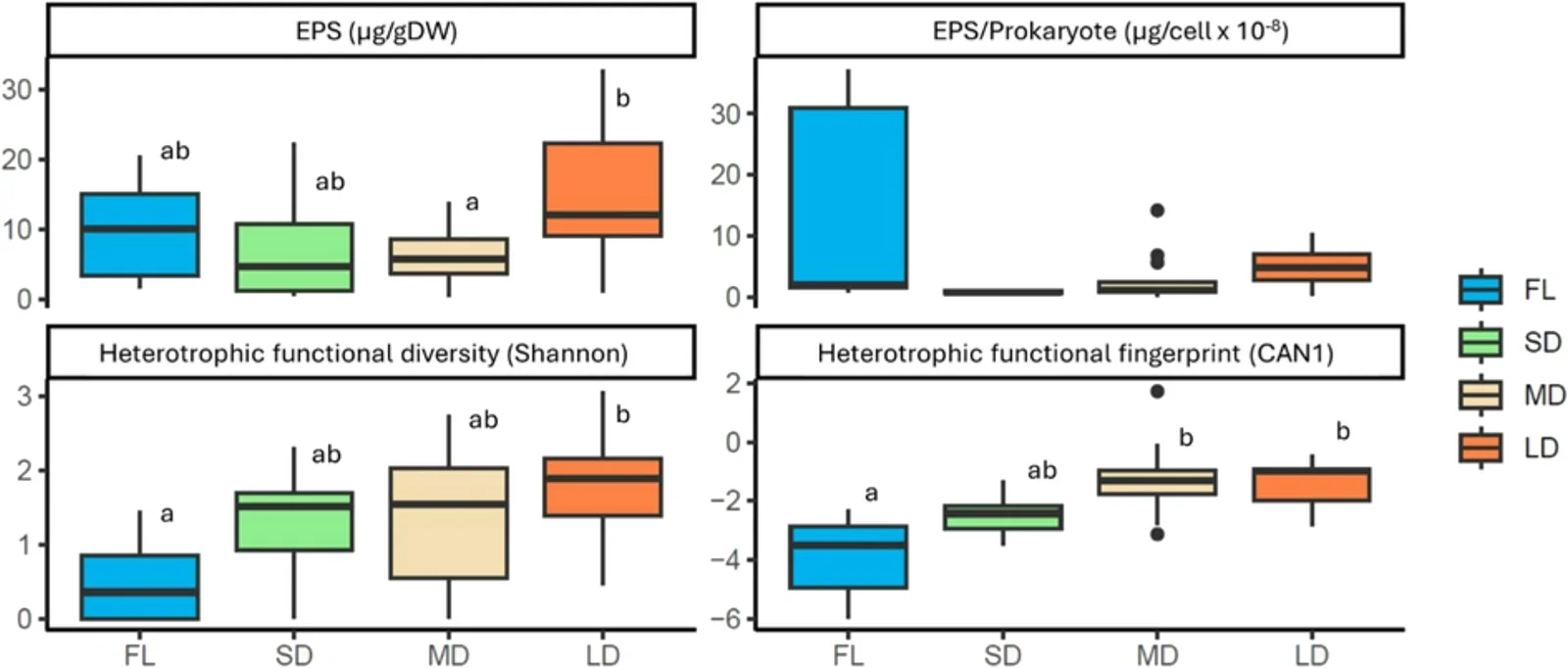
Boxplot across the four stream hydrological categories for: EPS (extracellular polymeric substances as the content of polysaccharides expressed in µg of glucose-equivalents per g sediment dry weight, DW); EPS per prokaryote cells, heterotrophic functional diversity (Shannon index after 24 h of microplate incubations), and heterotrophic functional fingerprint (CAN1, first axis of the canonical analysis, Fig. 1). Colours indicate the four stream hydrological categories: FL (Flow), SD (Short Dry), MD (Medium Dry), and LD (Long Dry). Low-case letters indicate significant different groups (*p* < 0.05) from post-hoc Tukey HSD test, after ANOVA analyses (Table 2)

**Fig. 3:**
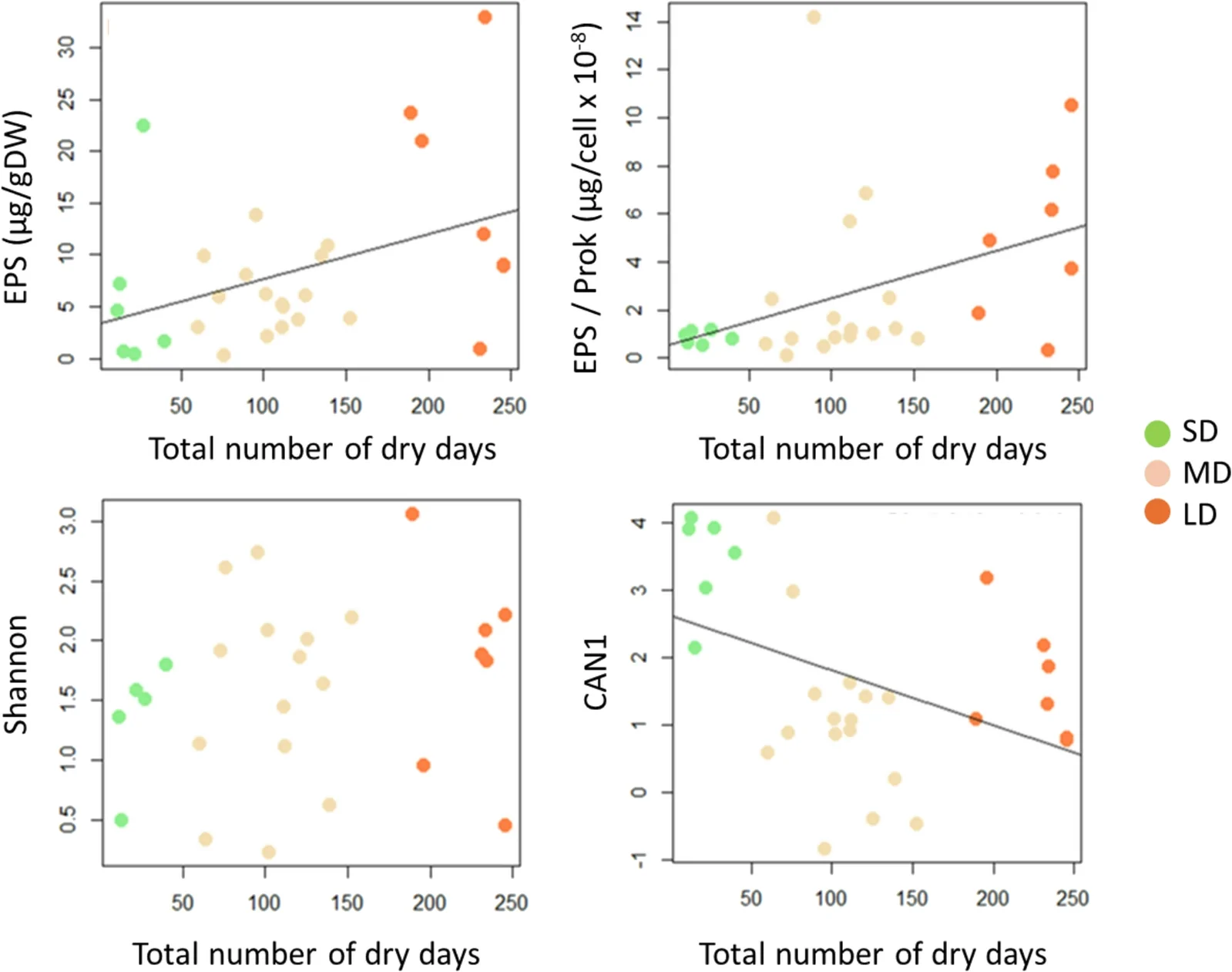
Relationships between the drought duration (number of dry days) and structural and functional responses of streambed biofilms. Structural and functional variables are represented by the following acronyms: EPS (Extracellular Polymeric Substances polysaccharide content), EPS/Prok (EPS per prokaryote cells), Shannon (heterotrophic functional diversity after 24 h of incubation at the Biolog Ecoplates), and CAN1 (first axis of the canonical analysis, representing the microbial functional fingerprint, Fig. 1). Colours indicate the three intermittent stream categories: SD (Short Dry period), MD (Medium Dry period), and LD (Long Dry period). The lines are plotted for the significant linear regressions (*p* < 0.05)

The prokaryotic density and chlorophyll-a content of the streambed biofilms did not show significant differences among hydrological categories (ANOVA, *p* > 0.05, Fig. S2). In contrast, EPS content and functional variables differed significantly across the four stream categories (Fig. 2; Table 2). EPS content was highest in LD streams compared to MD streams (Fig. 2; Table 2). When standardized per prokaryotic density and chlorophyll-a content, EPS did not show significant differences among hydrological categories but exhibited much higher variability in FL streams than in intermittent sites (Fig. 2, Fig. S2). Accordingly, when considering the entire dataset, no relationship was found between the drought duration (number of dry days) and EPS per prokaryotic density (EPS/prok, Suppl. Fig. S3) or chlorophyll content (EPS/chl). However, when FL streams were excluded from the analysis, a significant positive relationship emerged between the drought duration and EPS production, both in terms of total EPS content and EPS per prokaryotic density (linear regressions, R^2^_adj_ = 0.13, *p* = 0.03; R^2^_adj_ = 0.15, *p* = 0.02, respectively, Fig. 3).

**Table 2:** Results of one-factor ANOVA for the differences between stream hydrological categories for EPS (extracellular polymeric substances as polysaccharide content in µg of glucose-equivalents per g sediment dry Weight), EPS per prokaryote cells, heterotrophic functional diversity (Shannon index after 24 h of microplate incubations), and heterotrophic functional fingerprint (CAN1, first axis of the canonical analysis, Fig. 1). The F-Ficher and *p*-values are shown. Significance code is defined as: *** = *p* < 0.001, **= *p* < 0.01, *=*p* < 0.05,.=*p* < 0.1

	F-Ficher	*p*-value
EPS	2.977	0.0456*
EPS/Prokaryotes	1.213	0.3231
Shannon	3.005	0.0443*
CAN1	10.14	< 0.001***

Regarding biofilm function, heterotrophic functional diversity measured after 24 h microplate incubation (Shannon index) was higher at the driest sites than in FL streams (Fig. 2). A similar tendency was found for the heterotrophic functional diversity integrated for the whole incubation period but differences between stream hydrological categories were not statistically significant (Suppl Fig. S4). Additionally, the microbial functional fingerprint proxy (CAN1) significantly differed between FL sites and LD/MD sites (Fig. 2; Table 2). The drought duration showed significant positive linear relationship with both functional variables (linear regression, *p* = 0.03 for Shannon; *p* = 0.001 for CAN1; Fig. S3), indicating an increase in functional diversity and shifts in microbial carbon substrate utilization profile at drier sites. As FL sites were excluded, drought duration influenced microbial functional fingerprint proxy (CAN1, linear regression, R^2^_adj_ = 0.16, *p* = 0.019, Fig. 3) but not the functional diversity (Shannon). The relationship between functional and structural responses (CAN1 vs. EPS and EPS/prok) was also tested, both including and excluding FL sites, but no significant correlations were found (linear regressions, *p* > 0.05, Suppl Fig. S5).

## Discussion

This study highlights the critical role of EPS production in stream biofilms as an adaptive response to desiccation. The large-scale field approach provides strong evidence that EPS production is a fundamental microbial strategy for enhancing water retention and resilience to drought. These findings reinforce the theoretical framework of biofilm strong resistance and survival to harsh conditions thanks to its EPS matrix, which previously relied primarily on controlled laboratory experiments [34, 35]. Although field studies on stream biofilms suggest that microbial communities can adapt to desiccation by shifting their composition and functional traits, direct measurements of EPS production are still scarce and often inferred rather than quantified. For instance, Touchette et al. [36] showed in flume experiments beside a river that even short droughts (24 h) altered epilithic biofilm structure, indirectly suggesting enhanced EPS production as a protective mechanism. Similarly, mesocosm experiments in artificial channels simulating up to eight weeks of drought with phototrophic biofilms developing on polyethylene substrates revealed marked structural shifts under desiccation stress, but EPS measurements were not included [37]. Evidence from marine benthic systems also points in the same direction: sediment cores from a salt marsh exposed to 24 days of drought exhibited a significant increase in water-soluble carbohydrates, consistent with enhanced EPS production [38]. Altogether, these experimental observations across different habitats reinforce our field results, showing that EPS accumulation is a widespread microbial response to desiccation, although most controlled studies are limited to shorter drought periods and simplified laboratory communities compared to natural streambed biofilms. Unlike the experimental settings, this study examines natural biofilm communities, reflecting complex microbial assemblages and environmental interactions. Moreover, EPS production was observed to increase progressively in streambed biofilms with increasing drought stress, suggesting that microbial communities actively regulate EPS synthesis in response to the intensity and duration of desiccation. The relevance of this regulation response rather than of a change into a community with greater EPS production capability is supported by the non-significant differences in the prokaryotic communities’ composition between the different levels of intermittent sites [10].

Both autotrophic (eukaryotic and prokaryotic phototrophs, i.e. algae and cyanobacteria) and heterotrophic prokaryotes are well-known EPS producers. In marine benthic biofilms, diatoms produce EPS-polysaccharides, which facilitate desiccation resistance and support bacterial assemblages by enhancing nutrient cycling [39]. Similarly, cyanobacteria thrive in extreme environments partly due to their ability to form dense EPS matrices, playing key roles in soil biocrusts, microbial mats and phototrophic biofilms [40]. Microbial community composition can also influence EPS production. For example, Actinobacteria were found to be characteristic of intermittent streambeds [10], and their ability to produce EPS under hydration-desiccation cycles has been documented in desert biocrusts [41]. Also, species from the genera *Bacillus*, known as their typically biofilm formation capacity, are shown to increase EPS production under water stress [42]. Interestingly, EPS production was significantly correlated with drought when normalized per prokaryotic density but not per chlorophyll content. This suggests that heterotrophic prokaryotes, rather than algae, are the primary drivers of enhanced EPS secretion under desiccation stress. Note that although unicellular cyanobacteria may be included in both normalization approaches, they were little abundant in the studied sediment biofilms, as reported in [10]. These findings align with previous one showing that heterotrophic bacteria exhibit greater resistance to desiccation than algae and cyanobacteria [11, 12]. In intermittent streams it has been shown that autotrophic metabolism is highly sensitive to the duration and severity of non-flow periods, leading to drastic reductions in primary production [11, 12]. Furthermore, dominance of heterotrophic prokaryotes in EPS production might be linked to the nature of the studied biofilms, i.e. those developing on sediment (epipsammic). Epipsammic biofilms, specially from headwaters and typically forested streams, are commonly more dominated by heterotrophic organisms and display higher environmental sensitivity compared to the more autotrophic epilithic biofilms (growing on rocks) [43].

Despite the relationship between EPS production and drought, EPS content alone cannot be used as a universal indicator of water scarcity. In flowing streams, EPS per prokaryotic density varied widely across sites, suggesting that multiple environmental factors drive EPS production, including light availability, nutrient concentrations, hydrodynamics, and the composition of microbial communities [44–46]. On the other hand, in the intermittent streams, the EPS content per prokaryote cells showed a narrow variability between sites, although belonging to streams from different watersheds. This suggests that when intermittency occurs, drought is the crucial factor and the EPS production by prokaryotes is determined by the water availability conditions regardless biofilms originating from a large diversity of streambed sites and watersheds.

Beyond EPS production, functional fingerprinting clearly distinguished permanent from intermittent streams, indicating differences in organic matter utilization. While the microbial communities in permanently flowing streambeds preferentially utilized a polymer, a carboxylic acid and an amino acid compounds, intermittent streambed communities were characterized by a greater utilization of a phenolic compound as well as a use of a wider range of carboxylic acids and amino acids, and also of carbohydrates in the driest conditions (MD and LD sites). This pattern may suggest an adaptation to the availability of terrestrial organic matter sources which may become more prevalent in streams characterized by long-drought periods. Decaying plant material may provide phenolic compounds with aromatic rings such as lignin, and carbohydrates from complex polysaccharides (such as cellulose and hemicellulose). This aligns with increased phenol oxidase activity observed to be linked with increasing dryness in both field and laboratory studies [10, 16].

Contrary to our expectations, increasing drought did not reduce the diversity of carbon substrate utilization profiles. Given that EPS production is an energetically costly adaptation to drought, a reduction in microbial functions and functional diversity was initially expected. Results from controlled laboratory experiments which artificially induced drought conditions have shown that drought tends to reduce microbial functional diversity (e.g [47, 48]).,. In contrast, in the present study, intermittent sites exhibited higher functional diversity than permanent streams. This suggests that microbial communities in intermittent streambeds maintain functional diversity by broadening their capacity to metabolize diverse organic matter sources. This strategy may allow biofilms to exploit both aquatic and terrestrial carbon inputs, supporting ecosystem function despite fluctuating water availability. This observed opposite trend to those suggested from laboratory results, may indicate that our field approach captures the environmental long-term adaptation of streambed biofilms from the Mediterranean fluvial ecosystems. These in situ communities appear to possess inherent mechanisms that allow them to cope with intermittent water availability, highlighting the resilience of drought-adapted microbiomes [49]. As a one step further suggestion, given that the composition of prokaryotic communities differed between permanent flowing (FL) and intermittent sites, but showed no differentiation among the three intermittent categories and an increased abundance of soil-like taxa [10], it is conceivable that taxa inhabiting intermittent environments possess larger genomes, as described for terrestrial microbial communities [50]. This may confer a broader functional repertoire, potentially including genes involved in drought stress tolerance. This proposed pattern is consistent with observations that environments subject to high variability and resource scarcity select for microbes with more extensive genomic toolkits, while arid extremes favor smaller genomes [51]. This poses a broader ecological question as whether microbial communities in non-Mediterranean regions would be capable of developing similar adaptive traits in response to the increasing drought frequency predicted under future climate scenarios; and which would be the timescales of such adaptations occurring? As shown in the present work, from a wide range of intermittent Mediterranean streambeds where EPS production and a wide heterotrophic functional diversity appear as key microbial adaptive responses to environmental water scarcity. We could speculate that if drought duration periods enlarge, and the observed response mechanisms are maintained or even stressed, the overproduction of EPS can reduce the bacterial growth rate and the energy allocated to the EPS may compromise the rates of key ecosystem biogeochemical processes (i.e. nutrient and carbon cycling). Overall, these findings contribute to a better understanding of biofilm stress adaptation and the impacts of global change on aquatic ecosystems functioning. Furthermore, they offer valuable insight into the role of EPS in environmental resilience and its relationship to fluctuating environmental conditions.

## Supplementary Information

Below is the link to the electronic supplementary material.

Supplementary Material 1(PDF 705 KB)

## Data Availability

The datasets generated during and/or analysed during the current study are available from the corresponding author on reasonable request.

## References

[1] Flemming HC, Wuertz S (2019) Bacteria and archaea on earth and their abundance in biofilms. Nat Rev Microbiol 17:247–260. 10.1038/s41579-019-0158-9 30760902 10.1038/s41579-019-0158-9

[2] Battin T, Besemer K, Bengtsson M, Romaní AM, Packmann AI (2016) The ecology and biogeochemistry of stream biofilms. Nat Rev Microbiol 14:251–263. 10.1038/nrmicro.2016.15 26972916 10.1038/nrmicro.2016.15

[3] Mora-Gómez J, Freixa A, Perujo N, Barral-Fraga L (2016) Limits of the biofilm concept and types of aquatic biofilms. In: Romaní AM, Guasch H, Balaguer MD (eds) Aquatic biofilms: ecology, water quality and wastewater treatment. Caister Academic, pp 3–28

[4] Flemming HC, van Hullebusch ED, Little BJ, Neu TR, Nielsen PH, Seviour T, Stoodley P, Wingender J, Wuertz S (2025) Microbial extracellular polymeric substances in the environment, technology and medicine. Nat Rev Microbiol 23:87–105. 10.1038/s41579-024-01098-y 39333414 10.1038/s41579-024-01098-y

[5] Flemming HC, Wingender J, Szewzyk U, Steinberg P, Rice SA, Kjelleberg S (2016) Biofilms: an emergent form of bacterial life. Nat Rev Microbiol 14:563–575. 10.1038/nrmicro.2016.94 27510863 10.1038/nrmicro.2016.94

[6] Decho AW (2000) Microbial biofilms in intertidal systems: an overview. Cont Shelf Res 20:1257–1273. 10.1016/S0278-4343(00)00022-4

[7] Stevenson A, Cray JA, Williams JP, Santos R, Sahay R, Neuenkirchen N, McClure CD, Grant IR, Houghton JD, Quinn JP, Timson DJ, Patil SV, Singhal RS, Antón J, Dijksterhuis J, Hocking AD, Lievens B, Rangel DE, Voytek MA, Gunde-Cimerman N, Oren A, Timmis KN, McGenity TJ, Hallsworth JE (2015) Is there a common water-activity limit for the three domains of life? ISME J 9:1333–1351. 10.1038/ismej.2014.219 25500507 10.1038/ismej.2014.219 PMC4438321

[8] IPCC (Intergovernmental Panel on Climate Change) (2022) In: Pörtner HO, Roberts DC, Tignor M, Poloczanska ES, Mintenbeck K, Alegría A, Craig M, Langsdorf S, Löschke S, Möller V, Okem A, Rama B (eds) Impacts, Adaptation, and Vulnerability. Contribution of working group II to the sixth assessment report of the intergovernmental panel on climate change. Cambridge University Press, Cambridge, UK

[9] Tramblay Y, Koutroulis A, Samaniego L, Vicente-Serrano SM, Volaire F, Boone A, Le Page M, Llasat MC, Albergel C, Burak S, Cailleret M, Cindrić Kalin K, Davi H, Dupuy JL, Greve P, Grillakis M, Hanich L, Jarlan L, Martin-StPaul N, Martínez-Vilalta J, Mouillot F, Pulido-Velazquez D, Quintana-Seguí P, Renard D, Turco M, Türkeş M, Trigo R, Vidal JP, Vilagrosa A, Zribi M, Polcher J (2020) Challenges for drought assessment in the mediterranean region under future climate scenarios. Earth-Sci Rev 210,:103348. 10.1016/j.earscirev.2020.103348

[10] Gionchetta G, Artigas J, Arias-Real R, Oliva F, Romaní AM (2020) Multi-model assessment of hydrological and environmental impacts on streambed microbes in mediterranean catchments. Environ Microbiol 22:2213–2229. 10.1111/1462-2920.14990 32227440 10.1111/1462-2920.14990

[11] Timoner X, Acuña V, VonSchiller D, Sabater S (2012) Functional responses of stream biofilms to flow cessation, desiccation and rewetting. Freshwat Biol 57:1565–1578. 10.1111/j.1365-2427.2012.02818.x

[12] Colls M, Timoner X, Font C, Sabater S, Acuña V (2019) Effects of duration, frequency, and severity of the non-flow period on stream biofilm metabolism. Ecosystems 22:1393–1405. 10.1007/s10021-019-00345-1

[13] Jayathilake PG, Jana S, Rushton S, Swailes D, Bridgens B, Curtis T, Chen J (2017) Extracellular polymeric substance production and aggregated bacteria colonization influence the competition of microbes in biofilms. Front Microbiol 8:1865. 10.3389/fmicb.2017.01865 29021783 10.3389/fmicb.2017.01865 PMC5623813

[14] Nadell CD, Xavier JB, Levin SA, Foster KR (2008) The evolution of quorum sensing in bacterial biofilms. PLoS Biol 6(1):e14. 10.1371/journal.pbio.0060014 18232735 10.1371/journal.pbio.0060014 PMC2214811

[15] Gionchetta G, Frossard A, Bañeras L, Romani AM (2020) Changes in Precipitation Patterns: Responses and Strategies from Streambed Sediment and Soil Microbes. In: Marxsen J (ed) Climate Change and Microbial Ecology: Current Research and Future Trends (Second Edition). Caister Academic Press, UK, pp 391–420. 10.21775/9781913652579.12

[16] Gionchetta G, Oliva F, Menendez M, Lopez Laseras P, Romani AM (2019) Key role of streambed moisture and flash storms for microbial resistance and resilience to long-term drought. Freshwat Biol 64:306–322. 10.1111/fwb.13218

[17] Kakumanu ML, Ma L, Williams MA (2019) Drought-induced soil microbial amino acid and polysaccharide change and their implications for C-N cycles in a climate change world. Sci Rep 9:10968. 10.1038/s41598-019-46984-1 31358788 10.1038/s41598-019-46984-1 PMC6662807

[18] Artigas J, Fund K, Kirchen S, Morin S, Obst U, Romaní AM, Sabater S, Schwartz T (2012) Patterns of biofilm formation in two streams from different bioclimatic regions: analysis of microbial community structure and metabolism. Hydrobiologia 695:83–96. 10.1007/s10750-012-1111-3

[19] Sheremet A, Jones GM, Jarett J, Bowers RM, Bedard I, Culham C, Eloe-Fadrosh EA, Ivanova N, Malmstrom RR, Grasby SE, Woyke T, Dunfield PF (2020) Ecological and genomic analyses of candidate phylum WPS-2 bacteria in an unvegetated soil. Environ Microbiol 22:3143–3157. 10.1111/1462-2920.15054 32372527 10.1111/1462-2920.15054

[20] Freixa A, Ejarque E, Crognale S, Amalfitano S, Fazi S, Butturini A, Romaní AM (2016) Sediment microbial communities rely on different dissolved organic matter sources along a mediterranean river continuum. Limnol Oceanogr 61:1389–1405. 10.1002/lno.10308

[21] Zak JC, Willig MR, Moorhead DL, Wildman HG (1994) Functional diversity of microbial communities: a quantitative approach. Soil Biol Biochem 26:1101–1108

[22] Garland JL (1999) Potential and limitations of BIOLOG for microbial community analysis. Microbial biosystems: new frontiers. Proc 8th int symp microb ecol. Atlantic Canada Society for Microbial Ecology, Halifax, NS, pp 1–7

[23] Perujo N, Romaní AM, Martín-Fernández JA (2020) Microbial community-level physiological profiles: considering whole data set and integrating dynamics of colour development. Ecol Ind 117:106628. 10.1016/j.ecolind.2020.106628

[24] Pawlowsky-Glahn V, Egozcue JJ, Tolosana-Delgado R Compositional data and their sample space. In Modelling and Analysis of Compositional Data (V Pawlowsky-Glahn, Egozcue JJ, Tolosana-Delgado R (2015) 10.1002/9781119003144.ch2

[25] Egozcue JJ, Pawlowsky-Glahn V, Mateu-Figueras G et al (2003) Isometric logratio transformations for compositional data analysis. Math Geol 35:279–300. 10.1023/A:1023818214614

[26] Romaní AM, Fund K, Artigas J, Schwartz T, Sabater S, Obst U (2008) Relevance of polymeric matrix enzymes during biofilm formation. Microb Ecol 56:427–436. 10.1007/s00248-007-9361-8 18227962 10.1007/s00248-007-9361-8

[27] DuBois M, Gilles KA, Hamilton JK, Rebers PA, Smith F (1956) Colorimetric method for determination of sugars and related substances. Anal Chem 28:50–356. 10.1021/ac60111a017

[28] Jeffrey ST, Humphrey GF (1975) New spectrophotometric equations for determining chlorophylls a, b, c1 and c2 in higher plants, algae and natural phytoplankton. Bioch Physiol Pflanz 167(2):191–194

[29] Core Team R (2021) R: A language and environment for statistical computing. R Found Stat Comput. https://www.R-project.org/

[30] Palarea-Albaladejo J, Martín-Fernández JA (2015) zCompositions—R package for multivariate imputation of left-censored data under a compositional approach. Chemometr Intell Lab Syst 143:85–96. 10.1016/j.chemolab.2015.02.019

[31] Friendly M, Fox J (2017) Candisc: visualizing generalized canonical discriminant and canonical correlation analysis. R Package Version 0 8-0. 10.1016/S0167-9473(02)00290-6

[32] Fox J, Weisberg S (2019) An R companion to applied Regression, third edition. Sage, Thousand Oaks CA. https://www.john-fox.ca/Companion/

[33] Wickham H (2016) ggplot2: Elegant graphics for data analysis. Springer-Verlag New York. ISBN 978-3-319-24277-4. https://ggplot2.tidyverse.org

[34] Adessi A, Cruz de Carvalho R, De Philippis R, Branquinho C, da Silva JM (2018) Microbial extracellular polymeric substances improve water retention in dryland biological soil crusts. Soil Biol Biochem 116:67–69. 10.1016/j.soilbio.2017.10.002

[35] Üstüntürk-Onan M, Hoca S, Ilhan-Sungur E (2018) The effect of short-term drying on biofilm formed in a model water distribution system. Microbiology 87:857–864. 10.1134/S0026261718060188

[36] Touchette D, Gonzalez Mateu M, Michoud G, Deluigi N, Marasco R, Daffonchio D, Peter H, Battin T (2025) Experimental evidence on the impact of climate-induced hydrological and thermal variations on glacier-fed stream biofilms. FEMS Microbiol Ecol. 10.1093/femsec/fiae163 39674808 10.1093/femsec/fiae163 PMC11705997

[37] Barthès A, Ten-Hage L, Lamy A et al (2015) Resilience of aggregated microbial communities subjected to drought small-scale studies. Microb Ecol 70:9–20. 10.1007/s00248-014-0532-0 25403110 10.1007/s00248-014-0532-0

[38] McKew BA, Taylor JD, McGenity TJ, Underwood GJC (2011) Resistance and resilience of benthic biofilm communities from a temperate saltmarsh to desiccation and rewetting. ISME J 5(1):30–41. 10.1038/ismej.2010.91 20596071 10.1038/ismej.2010.91 PMC3105671

[39] Underwood GJC, Boulcott M, Raines CA, Waldron K (2004) Environmental effects on exopolymer production by marine benthic diatoms: dynamics, changes in composition, and pathways of production. J Phycol 40:293–304. 10.1111/j.1529-8817.2004.03076.x

[40] Rossi F, De Philippis R (2015) Role of cyanobacterial exopolysaccharides in phototrophic biofilms and in complex microbial mats. Life 5(2):1218–1238. 10.3390/life5021218 25837843 10.3390/life5021218 PMC4500136

[41] Baubin C, Ran N, Siebner H, Gillor O (2023) Divergence of biocrust active bacterial communities in the Negev desert during a hydration-desiccation cycle. Microb Ecol 86:474–484. 10.1007/s00248-022-02063-z 35788422 10.1007/s00248-022-02063-z

[42] Vardharajula S, Ali Z, Sk (2014) Exopolysaccharide production by drought tolerant Bacillus spp and effects on soil aggregation under drought stress. J Microbiol Biotech Food Sci; Nitra 4(1):51–57. 10.15414/jmbfs.2014.4.1.51-57

[43] Romaní AM, Sabater S (2001) Structure and activity of rock and sand biofilms in a mediterranean stream. Ecology 82(11):3232–3245. 10.1890/0012-9658(2001)082[3232:SAAORA]2.0.CO;2

[44] Pan M, Li H, Han X, Jiang S, Diao Y, Ma W, Li X, Qin J, Yao J, Wang Z (2023) Impact of hydrodynamic conditions on the production and distribution of extracellular polymeric substance in river biofilms. Water 15(21):3821. 10.3390/w15213821

[45] Proia L, Romaní AM, Sabater S (2012) Nutrients and light effects on stream biofilms: a combined assessment with CLSM, structural and functional parameters. Hydrobiologia 695:281–291. 10.1007/s10750-012-1117-x

[46] Chen Z, Xie Y, Qiu S, Li M, Ge S (2024) Enriched functional exoproteins and increased exopolysaccharides with altered molecular conformation mutually promoted indigenous microalgal-bacterial consortium biofilm growth under high light intensity. Chem Eng J 480:148056. 10.1016/j.cej.2023.148056

[47] Evans SE, Wallenstein MD (2014) Climate change alters ecological strategies of soil bacteria. Ecol Lett 17(2):155–164. 10.1111/ele.12206 24261594 10.1111/ele.12206

[48] Bouskill NJ, Chien Lim H, Borglin S, Salve R, Wood TE, Silver WL, Brodie EL (2013) Pre-exposure to drought increases the resistance of tropical forest soil bacterial communities to extended drought. ISME J 7(2):384–394. 10.1038/ismej.2012.113 23151641 10.1038/ismej.2012.113 PMC3554394

[49] Fierer N, Schimel JP, Holden PA (2003) Variations in microbial community composition through two soil depth profiles. Soil Biol Biochem 35(1):167–176. 10.1016/S0038-0717(02)00251-1

[50] Rodríguez-Gijón A, Nuy JK, Mehrshad M, Buck M, Schulz F, Woyke T, Garcia SL (2022) A genomic perspective across earth’s microbiomes reveals that genome size in archaea and bacteria is linked to ecosystem type and trophic strategy. Front Microbiol 12:761869. 10.3389/fmicb.2021.761869 35069467 10.3389/fmicb.2021.761869 PMC8767057

[51] Liu H, Zhang H, Powell J, Delgado-Baquerizo M, Wang J, Singh B. (2023) Warmer and drier ecosystems select for smaller bacterial genomes in global soils. Imeta. 3;2(1):e70. doi10.1002/imt2.70 38868347 10.1002/imt2.70 PMC10989973

