# Supplementary material for: Drought Drives Extracellular Polymeric Substances Accumulation and Functional Shifts in Streambed Biofilm Communities
Source: Microb Ecol. 2025 Nov 13;88(1):133. doi: 10.1007/s00248-025-02649-3 (PMC12647296; doi:10.1007/s00248-025-02649-3)
Supplement: Supplementary file 1 — Supplementary Material 1(PDF 705 KB) [file 248_2025_2649_MOESM1_ESM.pdf]

SUPPLEMENTARY MATERIALS

Drought drives extracellular polymeric substances accumulation and functional shifts in streambed biofilm communities

Romaní Anna M.[1], Perujo Núria[2], Pujol Marta[1], Gionchetta Giulia[3]

[1]GRECO, Institute of Aquatic Ecology, University of Girona, 17003 Girona, Spain

[2]Department of River Ecology, Helmholtz Centre for Environmental Research - UFZ, Brueckstrasse 3a, 39114 Magdeburg, Germany

[3]Department of Environmental Chemistry, Institute of Environmental Assessment and Water Research (IDAEA), Spanish Council of Scientific Research (CSIC), 08034 Barcelona, Spain

Corresponding author: anna.romani@udg.edu

Supplementary Information (SI)

SI1. Protocol for prokaryote density analysis

Prokaryote density from the sediment samples was measured following Amalfitano et al. (2009) and Perujo et al. (2016). Sediment samples were sonicated in cold for 1 min, shaken for 30 s, and sonicated again for 1 min to extract the biofilm from the sediment grains (sonication bath, Selecta, operating at 40W and 40 kHz). This soft 1+1 minute sonication (40W in a sonication bath, corresponding to ca. 5W of effective power to the sample) is within the power recommended of 1 minute at 20W of a sonication probe (effective power to the sample of ca. 10-15W) as a trade-off between cell detaching and cell lysis (Amalfitano and Fazzi, 2008). A subsample of the obtained extract (1 mL) was pipetted into a glass vial and 9 mL of detaching solution (distilled water, NaCl at 0.85%final concentration, Tween 20 at 0.5% final concentration and sodium pyrophosphate at 0.1 M, final concentration) was added to promote the separation and disaggregation of the cells (Amalfitano et al. 2008). The samples were shaken for 30 min (150 rpm, room temperature), left for 10 min at 4°C, and then sonicated with ice for two 1 min cycles. Finally, the samples were shaken for 1 min, and after 5 min to let large particles to settle down, 1 mL of supernatant was transferred into a clean sterile Eppendorf tube. The sediment extracts were then purified through Nycodenz (optiprep density gradient, Sigma-Aldrich, Merck KGaA, Darmstadt, Germany, 1 mL), added to the bottom of each Eppendorf tube, and centrifuged (14 000 rpm) for 90 min at 4°C. The purified sediment extracts (400 µL) were stained with Syto13 (4 µL, 5 µM solution, Thermo Fisher Scientific, Waltham, MA, USA) and incubated in the dark for 30 min. A beads solution was added as an internal pattern (10 µL of 10^6 beads·mL−1, Fisher 1.0 µm). Prokaryote density was determined by flow cytometry (FACSCalibur, Becton Dickinson, Franklin Lakes, NJ, USA). Results are given as cells per gram of sediment dry weight.

Amalfitano S, Fazi S, Puddu A (2009) Flow cytometric analysis of benthic prokaryotes attached to sediment particles. J Microbiol Methods 79(2):246–249. https://doi.org/10.1016/j.mimet.2009.09.005

Amalfitano S, Fazi S (2008). Recovery and quantification of bacterial cells associated with streambed sediments. J Microbiol Methods 75(2):237-243. https://doi.org/10.1016/j.mimet.2008.06.004.

Perujo N, Freixa A, Vivas Z, Gallegos AM, Butturini A, Romaní AM (2016) Fluvial biofilms from upper and lower river reaches respond differently to wastewater treatment plant inputs. Hydrobiol 765(1):169–183. https://doi.org/10.1007/s10750-015-2411-1

Supplementary Figures

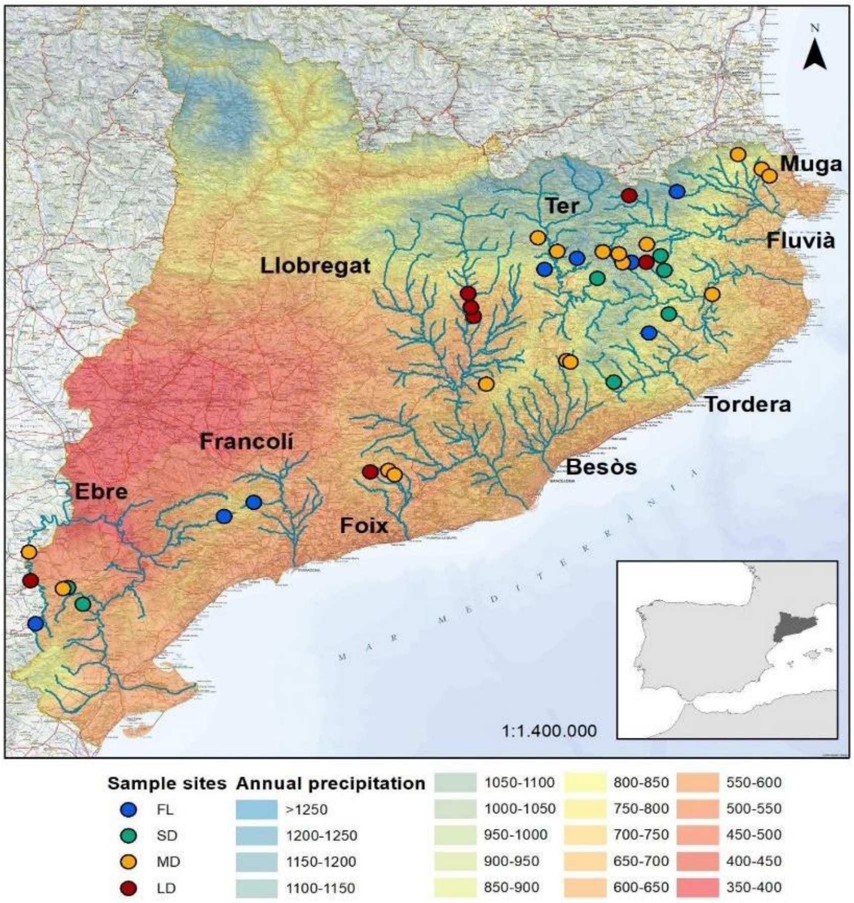

Fig. S1 Map showing the streambed sampling sites selected in Catalonia (ArcGIS v.10). The colour gradient represents the values of annual precipitation (www.icc.cat) and the sites' colour indicates the different sites' groups according to their hydrology (FL: Flow; SD: Short Dry; MD: Medium Dry; LD: Long Dry).

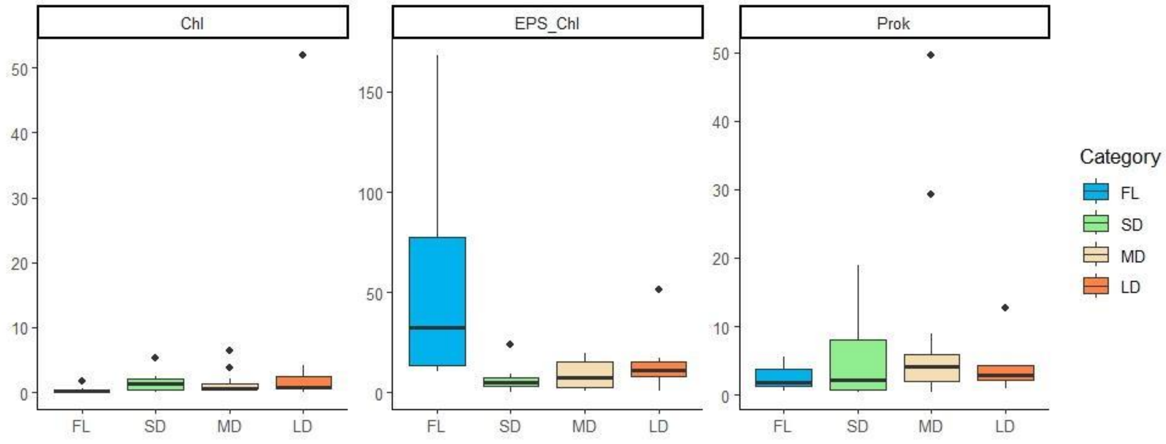

Fig. S2 Boxplot distribution of Chlorophyll-a content (Chl, µg/gDW), ratio of Extracellular polymeric substances per Chl content (EPS_Chl, µg/µg), and prokaryotes density (cell x 10^8/g DW) at the streambeds across the four stream categories. Colours indicate the four stream categories according to their hydrology: FL (Flow), SD (Short Dry), MD (Medium Dry), and LD (Long Dry)

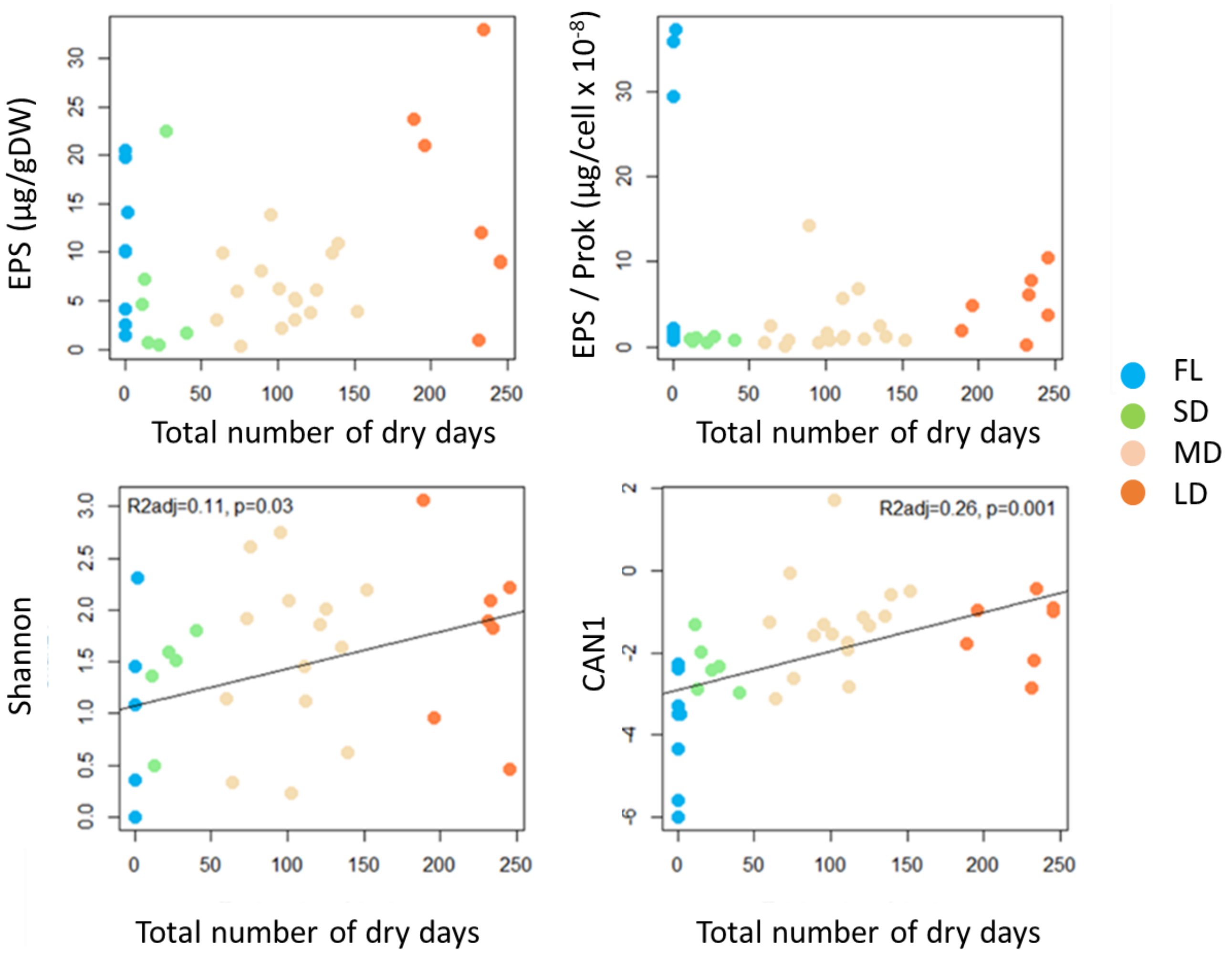

Fig. S3 Relationships between the drought duration (number of dry days) and structural and functional responses of streambed biofilms. Structural and functional variables are represented by the following acronyms: EPS (Extracellular Polymeric Substances polysaccharide content), EPS/Prok (EPS per prokaryote cells), Shannon (heterotrophic functional diversity after 24 h of incubation at the Biolog Ecoplates), and CAN1 (first axis of the canonical analysis, representing the microbial functional fingerprint, Fig. 1). Colours indicate the four stream categories according to their hydrology: FL (Flow), SD (Short Dry), MD (Medium Dry), and LD (Long Dry). The adjusted R^2 and *p*-values of significant linear regressions are displayed in each respective plot.

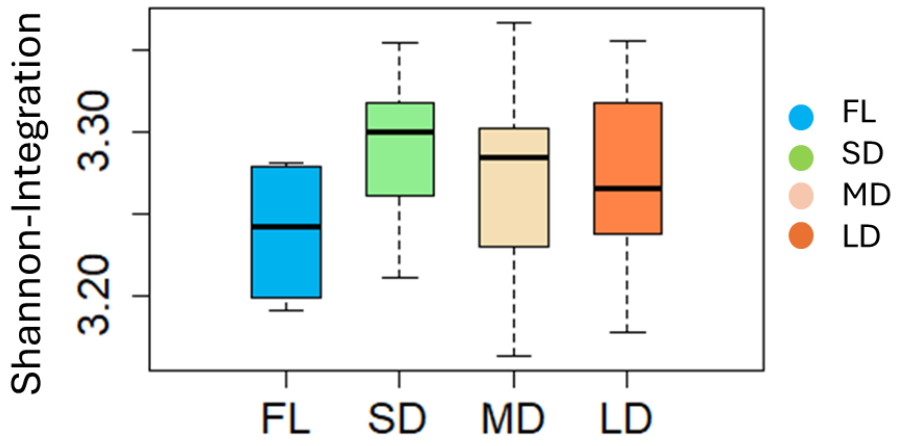

Fig. S4 Boxplot across the four stream hydrological categories for the Shannon index calculated by integrating the carbon susbtrate utilization results for the 6 days of incubation at the BiologEcoplates. Colours indicate the four stream hydrological categories: FL (Flow), SD (Short Dry), MD (Medium Dry), and LD (Long Dry).

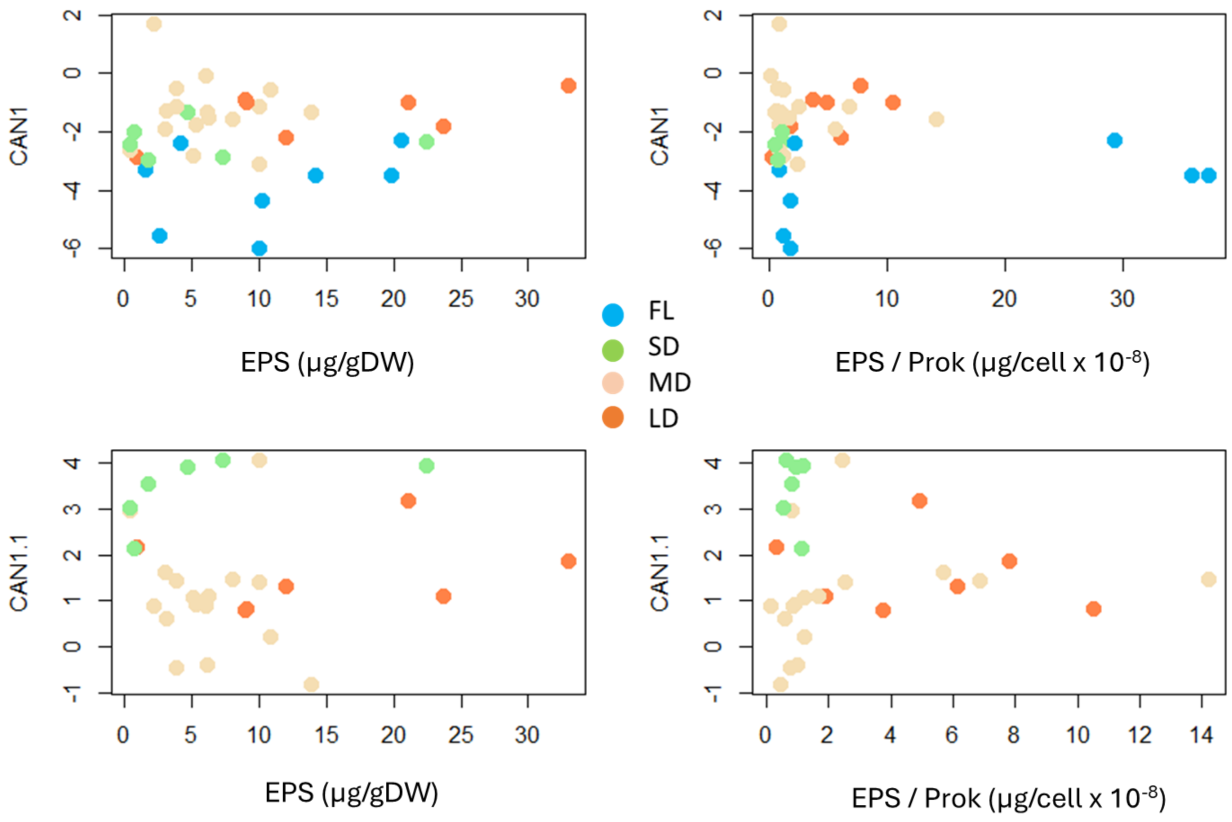

Fig. S5 Relationships between the CAN1 (first axis of the canonical analysis, representing the microbial functional fingerprint, Fig. 1) vs. EPS and EPS/prok content, including all sites (upper figures) and only intermittent sits (bottom figures). EPS (Extracellular Polymeric Substances polysaccharide content), EPS/Prok (EPS per prokaryote cells). Colours indicate the four stream categories according to their hydrology: FL (Flow), SD (Short Dry), MD (Medium Dry), and LD (Long Dry).

Table S1. List of the 31 carbon substrates present in the Biolog Ecoplates used to measure the Community level physiological profile and to obtain the proxy of the microbial functional fingerprint. The substrate abbreviation and the corresponding carbon source category (as described in Choi and Dobbs, 1999) are also included.

**Substrate name**	**Substrate abbreviation**	**Carbon source category**
Tween 40	Tw40	Polymer
Tween 80	Tw80	Polymer
alfa-cyclodextrin	α-Cycl	Polymer
Glycogen	Glyc	Polymer
D-cellobiose	D-Cell	Carbohydrate
alfa-D-Lactose	α-D-Lact	Carbohydrate
beta-methyl-D-glucoside	β-M-D-Gluc	Carbohydrate
D-Xylose	D-xyl	Carbohydrate
i-Erythritol	i-Er	Carbohydrate
D-Mannitol	D-Mann	Carbohydrate
N-Acetyl-D-glucosamine	N-A-D-Gluc	Carbohydrate
Glucose-1-Phosphate	G-1-P	Carbohydrate
D,L-alfa glycerol Phosphate	D,L-α-GlyPhos	Carbohydrate
D-Galactonic Acid gamma-Lactone	D-G-Lact	Carbohydrate
2-Hydroxy Benzoic Acid	2-HxBA	Phenolic compound
4-Hydroxy Benzoic Acid	4-HxBA	Phenolic compound
D-Galacturonic Acid	D-GalA	Carboxylic acid
Piruvic Acid Methyl Ester	PAME	Carboxylic acid
D-Glucosaminic Acid	D-GlucA	Carboxylic acid
Gamma-hydroxybutyric Acid	G-HxButA	Carboxylic acid
Itaconic Acid	ItcA	Carboxylic acid
alfa-ketobutyric Acid	α-KetA	Carboxylic acid
D-Malic Acid	D-MalA	Carboxylic acid
L-Arginine	L-Ar	Amino Acid
L-Asparagine	L-Asp	Amino Acid
L-Phenylalanine	L-Phe	Amino Acid
L-Serine	L-Ser	Amino Acid
L-Threonine	L-Thr	Amino Acid
Glycyl-L-Glutamic Acid	Glyc-L-GlutA	Amino Acid
Phenylethylamine	PA	Amine
Putrescine	Putr	Amine

Choi KH, Dobbs FC (1999) Comparison of two kinds of Biolog microplates (GN and ECO) in their ability to distinguish among aquatic microbial communities. J Microbiol Meth 36:203–213. doi:10.1016/S0167-7012(99)00034-2
